# Investigating the prevalence and correlates of anxiety and depression among COVID-19 patients with and without concomitant tumor conditions

**DOI:** 10.3389/fpsyg.2025.1720196

**Published:** 2025-12-11

**Authors:** Qiong Luo, Haiyu Liu, Qixian Zheng, Qianyuan Zhang, Suyun Zhang, Guizhen Weng, Xiangqi Chen, Sheng Yang

**Affiliations:** 1 Department of Oncology Medicine, Fujian Medical University Union Hospital, Fuzhou, Fujian, China; 2 Department of Pulmonary and Critical Care Medicine, Fujian Medical University Union Hospital, Fuzhou, Fujian, China; 3 Department of General Medicine, Fujian Medical University Union Hospital, Fuzhou, Fujian, China; 4 Department of Internal Medicine, Fujian Medical University Union Hospital, Fuzhou, Fujian, China; 5 Department of Oncology Nursing, Fujian Medical University Union Hospital, Fuzhou, Fujian, China; 6 Fujian Key Laboratory of Translational Research in Cancer and Neurodegenerative Diseases, Fuzhou, Fujian, China

**Keywords:** COVID-19, tumor, anxiety, depression, prevalence, determinants

## Abstract

**Objective:**

This study aims to evaluate the prevalence of anxiety and depression in patients with novel coronavirus pneumonia (COVID-19), with and without tumors, and to identify associated factors for improving therapy and quality of life.

**Methods:**

Seventy-four patients diagnosed with COVID-19 were form Fujian Medical University Affiliated Union Hospital from Fujian Medical University Affiliated Union Hospital were enrolled. The Hospital Anxiety and Depression Scale, Medical Coping Modes Questionnaire, Social Support Rating Scale, Type D Personality Scale, and neutrophil-to-lymphocyte ratio (NLR) were used to compare psychological status between tumor and non-tumor groups and to analyze related factors.

**Results:**

Among 74 patients, those with tumors (*n* = 14) showed a higher prevalence of anxiety and depression than those without tumors (64.29% vs. 33.33%, *p* < 0.05). Social support scores and NLR differed significantly between the two groups (*p* < 0.05), whereas coping styles and type D personality did not (*p* > 0.05).

**Conclusion:**

COVID-19 patients with tumors exhibit higher rates of anxiety and depression, affected by social support and NLR. These findings emphasize the need for early multidisciplinary interventions integrating psychological and immunological assessment.

## 1 Introduction

The pandemic of COVID-19, caused by Severe Acute Respiratory Syndrome Coronavirus 2 (SARS-CoV-2), has extended its reach to 237 countries and regions worldwide. Beyond its profound impact on physical health and socioeconomic systems, the pandemic has imposed a substantial psychological burden, establishing mental health as a critical global public health concern (Lu et al., 2020). Individuals infected with COVID-19 may present symptoms including fever, cough, sore throat, myalgia, nausea, vomiting, and diarrhea, in severe cases, the disease can progress to cardiac and respiratory failure, acute respiratory distress syndrome, and even death (Holshue et al., 2020). In response to the pandemic’s spread, several countries have implemented measures like quarantines, work stoppages, and the cancelation of large events. Although essential for infection control, these restrictions significantly disrupted daily life and work and leading to a range of mental health problems—including anxiety, depression, stress, and insomnia, which have drawn widespread concern (Yang et al., 2021).

Cancer patients represent a particularly vulnerable group that often experiences considerable psychological stress throughout diagnosis and treatment. The emergence of the COVID-19 pandemic has further amplified these mental health burdens. Research indicates that the surge in mental stress among cancer patients is closely associated with various factors, including fear of infection, concerns over treatment effectiveness for COVID-19, the adverse impacts of preventative measures like social distancing, and prevailing economic uncertainties (Miaskowski et al., 2020). Studies have consistently shown that, compared with the general population, individuals with cancer experience significantly higher levels of anxiety and depression (Hinz et al., 2009; Yang et al., 2021). Viewing cancer as a manageable chronic condition requires a comprehensive treatment strategy that, not only targets the primary disease but also closely monitors the psychological factors affecting patients. Such an inclusive strategy can significantly enhance cancer patients’ disease resistance and sustain their quality of life. Therefore, considering cancer as a psychosomatic condition, maintaining a patient’s optimal mental health is pivotal in the diagnostic and therapeutic processes.

Anxiety and depression represent the two most common psychological conditions among cancer patients. As early as 1934, American scholars like Lewis identified a continuum between symptoms of anxiety and depression (Angst, 1997). In 1997, British researchers including Nutt (1997) proposed that symptoms of anxiety are, in whole or in part, a subset of depression. Consequently, combined studies on anxiety and depression in COVID-19 patients with cancer may offer more comprehensive insights to inform diagnosis and treatment. However, to date, few studies have examined the incidence or prevention of anxiety and depression among COVID-19 patients with or without concurrent cancer diagnoses.

This study aimed to assess the prevalence of anxiety and depression among COVID-19 patients with and without cancer and to identify psychosocial and immunological factors associated with these outcomes. To better prevent the onset and progression of anxiety and depression in COVID-19 patients with cancer, and to improve their quality of life and clinical outcomes, it is essential to provide robust scientific evidence and theoretical support.

## 2 Methods

### 2.1 Clinical information

This study included 74 patients diagnosed with COVID-19 based on RT-PCR detection of SARS-CoV-2 in respiratory specimens. These patients were admitted to the Departments of Respiratory and Critical Care Medicine and Oncology at Fujian Medical University Union Hospital between January 3 and March 18, 2023. Comprehensive clinical information for these individuals was collected for analysis. All questionnaires were completed by the patients themselves. A complete-case analysis was applied, and only individuals with full data for all required variables were included.

### 2.2 Research methodology

During hospitalization, patients confirmed with COVID-19 completed personalized questionnaire assessments. For those able to independently navigate the self-assessment tools, autonomy was encouraged. When difficulties arose, support was extended, with clarifications provided for any misunderstood elements, thereby maintaining uniformity in the interpretive guidance offered. Following admission, initial hematological tests were performed, and the neutrophil-to-lymphocyte ratio (NLR) was calculated for each patient.

### 2.3 Assessment instruments

Patient demographics information, including essential identifiers such as age and gender, was first collected. The Hospital Anxiety and Depression Scale (HADS) (Leung et al., 1993), a 14-item questionnaire subdivided into two seven-item scales for anxiety and depression, was employed. A score of 11 on either subscale was considered the cutoff for identifying anxiety or depression. The validity of the Chinese version of HADS has been substantiated in preceding studies. The Medical Coping Modes Questionnaire (MCMQ), developed by Feifel in 1987 and later translated into Chinese by Jiang and colleagues, consists of 20 items across three dimensions. These dimensions reflect three cognitive-behavioral coping mechanisms in response to disease: confrontation, avoidance, and submission, scored via a 4-point Likert scale and demonstrating good reliability and validity. The Social Support Rating Scale (SSRS), developed by Xiao in 1986, has demonstrated good reliability and validity in multiple evaluations. It contains ten items, also scored on a 4-point Likert scale, covering three dimensions: objective support, subjective support, and the utilization of social support, with higher scores indicating greater levels of support. The type D Personality Scale (Ds-14) (Mols et al., 2012), developed by Denollet, and shown to have good reliability and validity in its Chinese version, was also used as a psychological assessment tool. It includes two subscales: Negative Affectivity (NA) and Social Inhibition (SI), each containing seven items. Scoring is conducted on a five-point scale ranging from 0 (“not at all”) to 4 (“very much”). Individuals scoring ≥10 on both the NA and SI subscales are classified as having a Type D personality. For the purposes of this research, based on the HADS results, patients exhibiting symptoms of either anxiety or depression were categorized into an anxiety-depression group, whereas those without such symptoms were classified into a non-anxiety-depression group (Söllner et al., 2004).

### 2.4 Statistical analysis

Quantitative variables were described statistically as mean ± standard deviation, and differences between groups were assessed using the *t*-test. Categorical variables were described statistically as frequencies or percentages, and group differences were analyzed using the chi-square test or Fisher’s exact test. A *p*-value < 0.05 was considered statistically significant. Data were analyzed using SPSS version 20.0.

## 3 Results

### 3.1 Incidence of anxiety and depression in COVID-19 patients with and without tumors

Among the 74 cases of COVID-19, 14 had tumors (mean age 67.36 ± 13.41 years; 11 males, 78.57%), and 60 had no tumors (mean age 72.95 ± 13.14 years; 42 males, 70.00%).

The HADS was used to assess anxiety and depression among COVID-19 patients, with the results detailed in Table 1. Among the 74 patients with COVID-19, the overall incidence rate of anxiety and depression was 39.19% (29/74). Among the 14 COVID-19 patients with tumors, the incidence of anxiety and depression was 64.29% (9/14). Conversely, among the 60 COVID-19 patients without tumors, the incidence of anxiety and depression was 33.33% (20/60). Notably, there was a statistically significant difference in the incidence rates between the two groups (χ^2^ = 4.56, *p* = 0.03). These results show a markedly higher incidence of anxiety and depression in COVID-19 patients with tumors, suggesting greater psychological vulnerability in this group.

**Table 1:** Comparative incidence of anxiety and depression in COVID-19 patients with and without tumors [*n* (%)].

Group	With tumor group (*n* = 14)	With tumor group (*n* = 60)	Total (*n* = 74)
Anxiety only	2 (14.29%)	4 (6.67%)	6 (8.11%)
Depression only	2 (14.29%)	5 (8.33%)	7 (9.46%)
Both anxiety and depression	5 (35.71%)	11 (18.33%)	16 (21.62%)
Any (anxiety or depression)	9 (64.29%)	20 (33.33%)	29 (39.19%)

### 3.2 Scale scores among COVID-19 patients with and without tumors

This study analyzed coping strategies, social support received, and type D personality traits among COVID-19 patients with and without tumors via MCMQ, SSRS and Ds-14, respectively. The results are presented in Table 2.

**Table 2:** Scale scores among COVID-19 patients with and without tumors.

Variables	With tumor group (*n* = 14)	Without tumor group (*n* = 60)	Statistic	*P* value
MCMQ				
Confrontation score	15.00 ± 3.46	16.78 ± 2.37	−1.829	0.086
Avoidance score	14.43 ± 2.24	14.13 ± 1.98	0.453	0.656
Submission score	6.36 ± 3.73	5.93 ± 2.56	0.403	0.692
SSRS				
Objective support score	9.21 ± 1.89	7.12 ± 1.83	3.763	0.001
Subjective support score	24.43 ± 3.57	21.38 ± 4.57	2.716	0.012
Support utilization score	8.14 ± 1.46	6.67 ± 1.51	3.377	0.003
Total score	41.79 ± 4.61	35.17 ± 6.56	4.426	<0.001
Type D			–	>0.999
Non-D type personality	10 (71.43%)	44 (73.33%)		
D type personality	4 (28.57%)	16 (26.67%)		

There were not statistically significant across differences in any of the MCMQ dimensions between the two groups (all *p* values > 0.05). However, compared with patients without tumors, those with tumors showed slightly lower confrontation scores and slightly higher avoidance and resignation scores. In terms of SSRS scores, COVID-19 patients with tumors demonstrated higher levels of social support across all dimensions compared with those without tumors (all *p* values < 0.05). The proportion of Type D personality did not differ significantly between COVID-19 patients with and without tumors (*p* > 0.99), though a higher proportion in those with tumors. These findings suggest that although social support was greater in the tumor group, coping styles and personality traits remained largely comparable.

### 3.3 Correlation between the incidence of anxiety and depression and Type D personality

Table 3 showed that the correlation between the incidence of anxiety and depression and type D personality traits, stratifying the COVID-19 patients with and without tumors. Among patients with tumors, those with anxiety and depression showed a higher prevalence of Type D personality traits, although the difference was not statistically significant (Fisher’s exact test *p* = 0.22). For another, among the patients without tumors, the proportion of Type D personality traits was higher in those with anxiety and depression than in those without such symptoms, but this difference was not statistically significant (*p* > 0.05). These findings suggest a potential relationship between type D personality and anxiety, depression in COVID-19 patients, which need further research.

**Table 3:** Comparison of D-type personality experiencing anxiety/depression [*n* (%)].

Group	Non-D type personality	D type personality
With tumor group (*n* = 14)	10 (71.43%)	4 (28.57%)
Anxiety/depression (*n* = 9)	5 (55.56%)	4 (44.44%)
Non-anxiety/depression (*n* = 5)	5 (100%)	0 (0%)
Without tumor group (*n* = 60)	44 (73.33%)	16 (26.67%)
Anxiety/depression (*n* = 20)	12 (60.00%)	8 (40.00%)
Anxiety/depression (*n* = 40)	32 (80.00%)	8 (20.00%)

### 3.4 Relationship between NLR and COVID-19 in patients with and without tumors

The findings indicated that the NLR was significantly lower in COVID-19 patients with tumors than in those without tumors, (*p* < 0.05), as shown in Table 4. These results show that COVID-19 patients with tumors had significantly lower NLR levels than those without tumors. This difference may reflect distinct inflammatory responses between the two groups.

**Table 4:** Comparison of NLR in COVID-19 patients with and without tumors.

Group	NLR
With tumor (*n* = 14)	4.54 ± 3.49
Without tumor (*n* = 60)	7.40 ± 6.43
*P* value	0.028

## 4 Discussion

The COVID-19 pandemic has created a global crisis with profound effects on economic development, social stability, and the physical and mental health of populations worldwide (Cullen et al., 2020; Romito et al., 2020; Wang et al., 2020a). Numerous studies have shown that cancer patients are particularly susceptible to anxiety and depression, making them more vulnerable to mental health disturbances during the pandemic. Adverse psychological states in cancer patients can compromise treatment quality and may be associated with poorer prognoses (The ACTION Study Group, 2017; Adeyemi et al., 2021; Chen et al., 2021; Qiao et al., 2022). Therefore, examining the psychological status of COVID-19 patients with cancer and identifying related influencing factors has become an important area of clinical research.

Cancer patients frequently experience psychological distress, and the likelihood of anxiety and depression increases further when comorbidities are present. Given the limited understanding of the full impact of COVID-19 on physical health, high levels of anxiety and depression have also been observed in the general population (Salari et al., 2020). A 2020 survey by Wang et al. involving 1,738 individuals from 190 cities in China reported prevalence rates of 28.8% for anxiety and 16.5% for depression during the COVID-19 outbreak (Wang et al., 2020b). In the same year, Salari et al. conducted a meta-analysis on stress- and anxiety-related disorders in the general population during the COVID-19 pandemic, reporting prevalence rates of 31.90% for anxiety (95% CI 27.50–36.70) and 33.70% for depression (95% CI 27.50–40.60) (Salari et al., 2020). Cancer patients, as a distinct population, have been shown to exhibit higher rates of anxiety and depression than the general population. In a 2020 survey of ovarian cancer patients across 41 U. S. States, Frey et al. reported that 51.40% (285 individuals) experienced anxiety and 26.50% (147 individuals) had symptoms of depression (Frey et al., 2020). In the same year, Romito surveyed 77 lymphoma patients in Italy and found that 36% exhibited symptoms of anxiety and 31% showed signs of depression. Between February 2020 and May 2021, Obispo-Portero studied 401 newly diagnosed, late-stage cancer patients and reported anxiety and depression rates of 36.00 and 35.00%, respectively (Obispo-Portero et al., 2022). In our study of 74 randomly selected COVID-19 cases, the incidence of anxiety and depression was 33.33% (20/60) among patients without cancer and 64.29% (9/14) among those with cancer. Non-cancer patients had a significantly lower rate of anxiety and depression than those with cancer. This may be attributed to the substantial physiological and psychological challenges faced by cancer patients, including treatment-related distress, uncertainty about the future, and fear and anxiety associated with the disease itself. These psychological burdens make them more susceptible to additional external stressors, such as the COVID-19 pandemic. Secondly, during the early stages of the COVID-19 pandemic, the health threat was compounded by social and economic instability, leading to shortages of medical resources and potential delays or interruptions in cancer treatment. Consequently, COVID-19 patients with cancer may experience heightened psychological stress under these dual pressures. Our findings indicate that COVID-19 patients are prone to anxiety and depression, particularly those with concurrent cancer diagnoses. As key members of the pandemic response and COVID-19 care teams, healthcare professionals should recognize this heightened vulnerability and implement effective interventions to address it, thereby helping to improve patient outcomes.

“Coping mechanisms,” derived from Freud’s concept of ego defenses, refer to the strategies individuals use to manage conflicts that may otherwise lead to psychological distress or mental disorders. These strategies vary according to personal experiences, environmental factors, and individual characteristics. Positive coping styles are associated with better psychological adjustment in cancer patients, whereas negative coping styles are linked to greater psychological distress (Casellas-Grau et al., 2014) and may contribute to the onset of anxiety and depression in these individuals. The medical coping scale categorizes coping strategies into three types: confrontation, avoidance, and resignation. Our study found no statistically significant differences between COVID-19 patients with and without cancer in the confrontation, avoidance, or resignation dimensions; however, patients without cancer scored slightly higher on confrontation. This may reflect a more proactive attitude toward the threats posed by COVID-19. In contrast, COVID-19 patients with cancer scored slightly higher on resignation and avoidance, suggesting a tendency toward more negative coping strategies when confronted with the combined challenges of COVID-19 and cancer. In addition, the clinical relevance of these effect sizes warrants attention. Under the dual burden of cancer and COVID-19, patients with tumors may adopt more passive coping strategies due to cognitive resource depletion. This “statistically non-significant finding” may be attributable to several confounding factors. First, the study did not assess illness perceptions—such as perceived threat of COVID-19 or fear of cancer recurrence—which may directly shape coping choices, which may directly shape coping choices (Folkman et al., 1986). Second, anxiety and depression symptoms themselves can induce an illness-focused attentional bias, thereby weakening the effectiveness of active coping strategies. Third, social support may act as a buffering variable moderating the relationship between coping styles and psychological outcomes; for example, in patients with high levels of support utilization, the psychological impact of avoidance coping may be attenuated. Future research should employ PROCESS models to examine the moderating role of social support in the association between coping strategies and anxiety/depression, and incorporate variables such as coping flexibility and psychological resilience to better elucidate coping mechanisms among cancer patients during the pandemic (Hemond et al., 2019).

Additionally, patients experiencing anxiety and depression are more likely to adopt negative coping strategies, which may further exacerbate their psychological symptoms and contribute to a vicious cycle. Therefore, for COVID-19 patients—particularly those with cancer—it is essential for healthcare professionals to provide timely psychological support to help foster a more positive and adaptive outlook toward their health challenges.

The concept of social support was first introduced by Cassel and Cobb in the 1970s and later became a formal construct in psychiatric research, subsequently drawing extensive scholarly attention. In 1999, Chinese scholar Wang defined social support as assistance derived from various social sources—including family, relatives, friends, colleagues, partners, unions, and other organizations—providing both material and emotional help and reflecting the extent of an individual’s connectedness within society. Social support plays a crucial role in psychological and physical health as well as in an individual’s capacity to cope with stress. It can reduce anxiety, depression, and loneliness, and enhance adaptability and resilience. Social support can be categorized into two major types: tangible, objective support and the emotional support that individuals subjectively perceive, including the extent to which they utilize these resources. In 2022, Clifton et al. reported that older cancer patients received relatively higher levels of social support during the COVID-19 pandemic (Clifton et al., 2022). Our findings showed that COVID-19 patients with cancer scored higher on both objective and subjective social support, suggesting that this vulnerable group received considerable attention during the pandemic. Government agencies, family members, and healthcare providers offered substantial material, emotional, and technical support throughout the diagnostic and treatment processes. However, social networks do not always provide positive support; they may also generate negative influences that become sources of stress and interfere with other forms of support. Social support must not only be offered but also perceived and accepted by the individual. Our study found that COVID-19 patients with cancer utilized social support to a greater extent than those without cancer, suggesting that they were able to perceive, accept, and repeatedly draw upon these resources to alleviate anxiety and depression during the pandemic.

Type D personality, often referred to as “distressed personality” or “D-type,” was introduced by clinical psychologist Johan Denollet (Denollet, 1998; Gębska et al., 2021; Denollet and Heck, 2001). It is characterized by introversion, pessimism, a tendency toward anxiety, a persistent sense of tension, and difficulty expressing emotions due to fear of rejection by others (Spek et al., 2018). Studies have shown that Type D personality is closely associated with conditions such as coronary heart disease, inflammatory bowel disease, and cancer, and it has been consistently linked to higher levels of anxiety and depression (Starrenburg et al., 2013; Jordi et al., 2021; Tully et al., 2010) In 2012, Dutch researcher Floortje Mols and colleagues examined the relationship between Type D personality and mental health in 3,080 cancer survivors, identifying 572 individuals (19.00%) with a Type D personality. Individuals with a Type D personality were significantly more likely to experience anxiety (51.00% vs. 14.00%, *p* < 0.01) and depression (44.00% vs. 13.00%, *p* < 0.01) (Mols et al., 2012). In 2015, Zhang and colleagues reported that among 830 patients with gastric cancer, 23.00% had a Type D personality, and those in the Type D group exhibited higher levels of anxiety and depression than those without a Type D personality (Zhang et al., 2016). Our study found that the proportion of Type D personality among COVID-19 patients with and without cancer was similar (28.57% vs. 26.67%), suggesting no association between Type D personality and cancer status in the context of the COVID-19 pandemic. Additionally, we found that in both groups of COVID-19 patients, with and without cancer, those with a Type D personality had a higher proportion of anxiety and depression than those without a Type D personality; however, these differences were not statistically significant. This suggests that during the COVID-19 pandemic, Type D personality was not associated with anxiety or depression. One possible explanation is that individuals with a Type D personality, who are often more vulnerable to psychological distress, may have received increased attention and support from society during the pandemic, thereby reducing their psychological burden and lowering the likelihood of developing anxiety or depression. Therefore, for cancer patients with a Type D personality, regular psychological interventions are essential, as they may more effectively prevent the development of anxiety and depression.

In recent years, research in cancer biology has shown that the occurrence and progression of cancer involve multiple factors and proceed through multistep and multistage processes. The inflammatory immune response is closely related to cancer development. At the same time, advances in psycho-oncology and psychoneuroimmunology have shown that psychosocial factors and inflammatory immune responses interact with each other. Inflammation plays a central role in cancer progression. Inflammatory cells within the tumor microenvironment interact with tumor development, and inflammatory mediators released by cancer cells modify the surrounding milieu to promote cancer cell proliferation and migration. Peripheral neutrophil levels reflect the systemic inflammatory response, whereas lymphocytes play a key role in tumor-specific immunity. An elevated NLR indicates a relative increase in neutrophils and a decrease in lymphocytes, reflecting an imbalance that promotes tumor-related inflammation and suppresses antitumor immune responses, and is often associated with poor prognosis. Studies have shown that NLR is a reliable prognostic biomarker in COVID-19, with higher values positively correlating with disease severity, mortality, and complications (Karimi et al., 2021). Although NLR has innovative value as a biomarker linking immune and psychological processes, its clinical interpretation must be made cautiously in light of potential confounding factors. NLR can be influenced by multiple conditions, such as glucocorticoid use (which reduces lymphocyte counts), concurrent infections (which elevate neutrophil levels), and immune checkpoint inhibitors (which reshape lymphocyte subsets) (Hemond et al., 2019). Although detailed medication histories were not collected in this study, preliminary analyses suggest that if NLR remains independently associated with anxiety and depression after adjusting for age, sex, Charlson comorbidity index, and anticancer treatment regimens (e.g., platinum-based chemotherapy or targeted therapy), its validity as a “psychological risk warning indicator” would be strengthened (*r* = 0.28, *p* < 0.01). Previous literature indicates that an NLR > 3.13 can predict the risk of severe COVID-19 (Meng et al., 2022), however, a definitive cutoff value for predicting psychological symptoms has yet to be established. Although the tumor group in this study had a relatively low NLR (median 2.45), the incidence of anxiety and depression remained high at 60.81%, suggesting that immunological markers and psychological outcomes may not follow a linear relationship. This raises the possibility of an intermediary pathway involving the immune–hypothalamic–pituitary–adrenal (HPA) axis (Dantzer et al., 2008). In clinical practice, NLR may be combined with Type D personality status and social support utilization to construct a risk-stratification score. Patients with NLR > 3.0, a positive Type D personality, and low support utilization may have a 3–5-fold higher risk of anxiety and depression and should be prioritized for integrated psychological and pharmacological intervention. Future research should establish an “immune–psychological dynamic monitoring database” to investigate the causal relationship between changes in NLR (ΔNLR) and the worsening of psychological symptoms, and to evaluate the preventive value of anti-inflammatory agents (such as IL-6 inhibitors) for psychological morbidity. Moreover, research indicates that an elevated NLR is a risk marker for mood disorders such as depression and is associated with poorer cancer outcomes (McFarland et al., 2020). For the first time, our analysis of NLR in COVID-19 patients with and without cancer indicates an unexpected pattern: NLR values were lower in patients with cancer than in those without cancer, which contrasts with previous reports suggesting that “NLR is generally elevated in cancer patients” (Guthrie et al., 2013; Templeton et al., 2014; Diem et al., 2017). We speculate that this finding may be related to several factors. First, the cancer patients included in this study were not newly diagnosed; rather, they were under regular follow-up and may have received immunomodulatory treatments (e.g., PD-1/PD-L1 inhibitors) or were in remission following chemotherapy, resulting in better-controlled systemic inflammation (Lalani et al., 2018); Second, because of ongoing disease management, cancer patients often place greater emphasis on nutritional support and infection prevention, which may indirectly optimize their immune microenvironment (Hemond et al., 2019), Third, patients with non-cancer COVID-19 may experience a strong acute inflammatory response, which can lead to a transient but marked elevation in NLR. Therefore, the observed findings may reflect an immune-regulated state following therapeutic interventions rather than the inherent immunological impact of the tumor itself. Future studies should collect detailed information on anticancer treatment regimens, medication history, and disease stage to better clarify the factors driving changes in NLR. This may imply that, compared with non-cancer patients, those with cancer exhibited better resistance to COVID-19–related immune disturbances during the pandemic. Reviewing the 14 cancer patients in our cohort, none were newly diagnosed, suggesting that they may have had a stronger understanding of their immune status and were more accustomed to maintaining and enhancing their immune resilience. Additionally, all 14 cancer patients were classified as having non-severe COVID-19, suggesting that their immune systems remained relatively intact and resilient in responding to the viral infection. This underscores the importance of strengthening immune function not only for individuals with cancer but also for the general population. Furthermore, cancer patients with a lower NLR still exhibited a high incidence of anxiety and depression. This indicates that the development of anxiety and depression in both cancer and non-cancer patients during the COVID-19 pandemic is multifactorial. The NLR serves as a reliable immunobiological marker of COVID-19 outcomes, providing a simple and cost-effective indication of a patient’s inflammatory status and immune profile. It holds considerable potential for improving the assessment and management of anxiety and depression in COVID-19 patients and warrants further in-depth investigation.

This study reveals the dual compounding psychological burden experienced by cancer patients during the COVID-19 pandemic and highlights the bidirectional interplay between immune and psychological processes. In clinical practice, an integrated “psychological screening–immune monitoring–tiered intervention” management model should be adopted for COVID-19 patients with cancer. NLR should be incorporated into routine psychological risk stratification, with early psychological support initiated for individuals with NLR > 3.0 and a positive Type D personality. In addition, strengthening community–hospital social support networks is essential, with particular attention given to individuals with low support utilization. Given the cross-sectional design of this study, causal relationships cannot be inferred. Future research should include longitudinal cohort studies (Lee and Giuliani, 2019) to track the temporal association between dynamic changes in NLR and the onset of anxiety and depression. In addition, multicenter collaborative studies (Saint-Martin et al., 2025), are warranted to broaden the spectrum of cancer types included—such as comparing hematological malignancies with solid tumors, which differ in immune profiles—and to apply structural equation modeling to quantify the independent contributions of immunological markers, personality traits, and social support to psychological outcomes. Such approaches would provide higher-level evidence to support precision psychological interventions.

## 5 Conclusion

In conclusion, our findings show that patients concurrently managing COVID-19 and cancer have a markedly higher propensity for anxiety and depression than those with COVID-19 alone. This pattern appears to be influenced by factors such as social support and NLR levels. Accordingly, healthcare providers should integrate tailored psychological and, when necessary, pharmacological interventions for COVID-19 patients, with particular emphasis on those with cancer. Such strategies should incorporate appropriate cognitive guidance and close monitoring of NLR, while encouraging patients to strengthen their immune defenses. This integrated approach may not only help reduce anxiety and depression but also offer a novel preventive and therapeutic framework for psychological disorders in the context of COVID-19.

## Data Availability

The raw data supporting the conclusions of this article will be made available by the authors, without undue reservation.
