# Correction: Investigating the prevalence and correlates of anxiety and depression among COVID-19 patients with and without concomitant tumor conditions

**DOI:** 10.3389/fpsyg.2026.1796560

**Published:** 2026-02-25

**Authors:** Qiong Luo, Haiyu Liu, Qixian Zheng, Qianyuan Zhang, Suyun Zhang, Guizhen Weng, Xiangqi Chen, Sheng Yang

**Affiliations:** 1 Department of Oncology Medicine, Fujian Medical University Union Hospital, Fuzhou, Fujian, China; 2 Department of Pulmonary and Critical Care Medicine, Fujian Medical University Union Hospital, Fuzhou, Fujian, China; 3 Department of General Medicine, Fujian Medical University Union Hospital, Fuzhou, Fujian, China; 4 Department of Internal Medicine, Fujian Medical University Union Hospital, Fuzhou, Fujian, China; 5 Department of Oncology Nursing, Fujian Medical University Union Hospital, Fuzhou, Fujian, China; 6 Fujian Key Laboratory of Translational Research in Cancer and Neurodegenerative Diseases, Fuzhou, Fujian, China

**Keywords:** COVID-19, tumor, anxiety, depression, prevalence, determinants

An incorrect **Funding** statement was provided. No funding was received for this work. The corrected **Funding** statement appears below:

“The author(s) declared that financial support was not received for this work and/or its publication.”

The original version of this article has been updated.

